# Cell lineage of timed cohorts of *Tbx6*-expressing cells in wild-type and *Tbx6* mutant embryos

**DOI:** 10.1242/bio.026203

**Published:** 2017-06-12

**Authors:** Daniel Concepcion, Andrew J. Washkowitz, Akiko DeSantis, Phillip Ogea, Jason I. Yang, Nataki C. Douglas, Virginia E. Papaioannou

**Affiliations:** 1 Department of Genetics and Development, Columbia University Medical Center, New York, NY 10032, USA; 2 Department of Obstetrics and Gynecology, Divisions of Reproductive Sciences and Reproductive Endocrinology and Infertility, Columbia University Medical Center, New York, NY 10032, USA

**Keywords:** *Tbx6*, T-box, Mutant phenotype, Cell fate, Lineage, Mouse

## Abstract

Tbx6 is a T-box transcription factor with multiple roles in embryonic development as evidenced by dramatic effects on mesoderm cell fate determination, left/right axis determination, and somite segmentation in mutant mice. The expression of *Tbx6* is restricted to the primitive streak and presomitic mesoderm, but some of the phenotypic features of mutants are not easily explained by this expression pattern. We have used genetically-inducible fate mapping to trace the fate of *Tbx6*-expressing cells in wild-type and mutant embryos to explain some of the puzzling features of the mutant phenotype. We created an inducible Tbx6-creERT2 transgenic mouse in which *cre* expression closely recapitulates endogenous *Tbx6* expression both temporally and spatially. Using a lacZ-based Cre reporter and timed tamoxifen injections, we followed temporally overlapping cohorts of cells that had expressed *Tbx6* and found contributions to virtually all mesodermally-derived embryonic structures as well as the extraembryonic allantois. Contribution to the endothelium of major blood vessels may account for the embryonic death of homozygous mutant embryos. In mutant embryos, Tbx6-creERT2-traced cells contributed to the abnormally segmented anterior somites and formed the characteristic ectopic neural tubes. Retention of cells in the mutant tail bud indicates a deficiency in migratory behavior of the mutant cells and the presence of Tbx6-creERT2-traced cells in the notochord, a node derivative provides a possible explanation for the heterotaxia seen in mutant embryos.

## Abstract (precis)

**Summary:** Embryonic cells that transiently express the transcription factor, Tbx6, during the process of gastrulation have been tracked in later development in wild-type and *Tbx6* homozygous mutant embryos, where they give rise to the ectopic neural tubes characteristic of the mutant phenotype.

## INTRODUCTION

The T-box transcription factor gene *Tbx6* is a critical gene for the determination and differentiation of mesoderm during gastrulation. Embryos homozygous for a null mutation die at midgestation with multiple hematomas, abnormally segmented rostral somites, ectopic neural tubes in place of the more caudal somites, an enlarged tail bud, and heterotaxia (Chapman et al., 1996; Chapman and Papaioannou, 1998; Hadjantonakis et al., 2008). Embryos with reduced levels of Tbx6 are viable and lack ectopic neural tubes, but have severe defects in somite patterning and differentiation (Watabe-Rudolph et al., 2002; White et al., 2003). A *TBX6* mutation in humans results in spondylocostal dysostosis (Sparrow et al., 2013). Although *Tbx6* has a limited expression pattern in the primitive streak, presomitic mesoderm and tail bud of the mouse embryo during mesoderm ingression and somitogenesis, the effects of mutations are quite diverse due to the multiple cell fates of early mesoderm, which have been well established by classical fate-mapping studies (Beddington, 1994; Lawson et al., 1991; Smith et al., 1994; Sulik et al., 1994; Tam and Beddington, 1987; Wilson and Beddington, 1996). However, because the lines drawn in fate-mapping studies are imprecise, it is not known exactly where cells that express *Tbx6* are bound in later development, how this affects the mutant phenotype, or how mutation of *Tbx6* affects cell lineage.

To answer these questions, we traced the lineage of cells that have expressed *Tbx6* using genetically-inducible fate mapping (GIFM). This method provides additional information to traditional fate mapping by tracing the fate of cells that express a particular gene of interest (Joyner and Zervas, 2006). GIFM is accomplished by crossing mice carrying a transgene with a specific promoter driving the expression of *cre* recombinase with mice carrying a loxP-flanked reporter transgene that allows the visualization of cells that express Cre. Because the Cre-induced recombination event in the reporter transgene is irreversible and heritable, the reporter tracks the fate of the cells whether or not they continue to express *cre*. By using a tamoxifen-inducible *cre* transgene, this technique can be used to mark cohorts of cells that express *cre* during different developmental intervals. In this study we have produced a transgenic mouse with *cre* expressed in the *Tbx6* expression domain and have used this mouse to trace the lineage of cells that express *Tbx6* at some point in their history in both wild-type and *Tbx6* homozygous mutant embryos.

## RESULTS

### *Tbx6* expression-reporter transgene

**Fig. 1.**
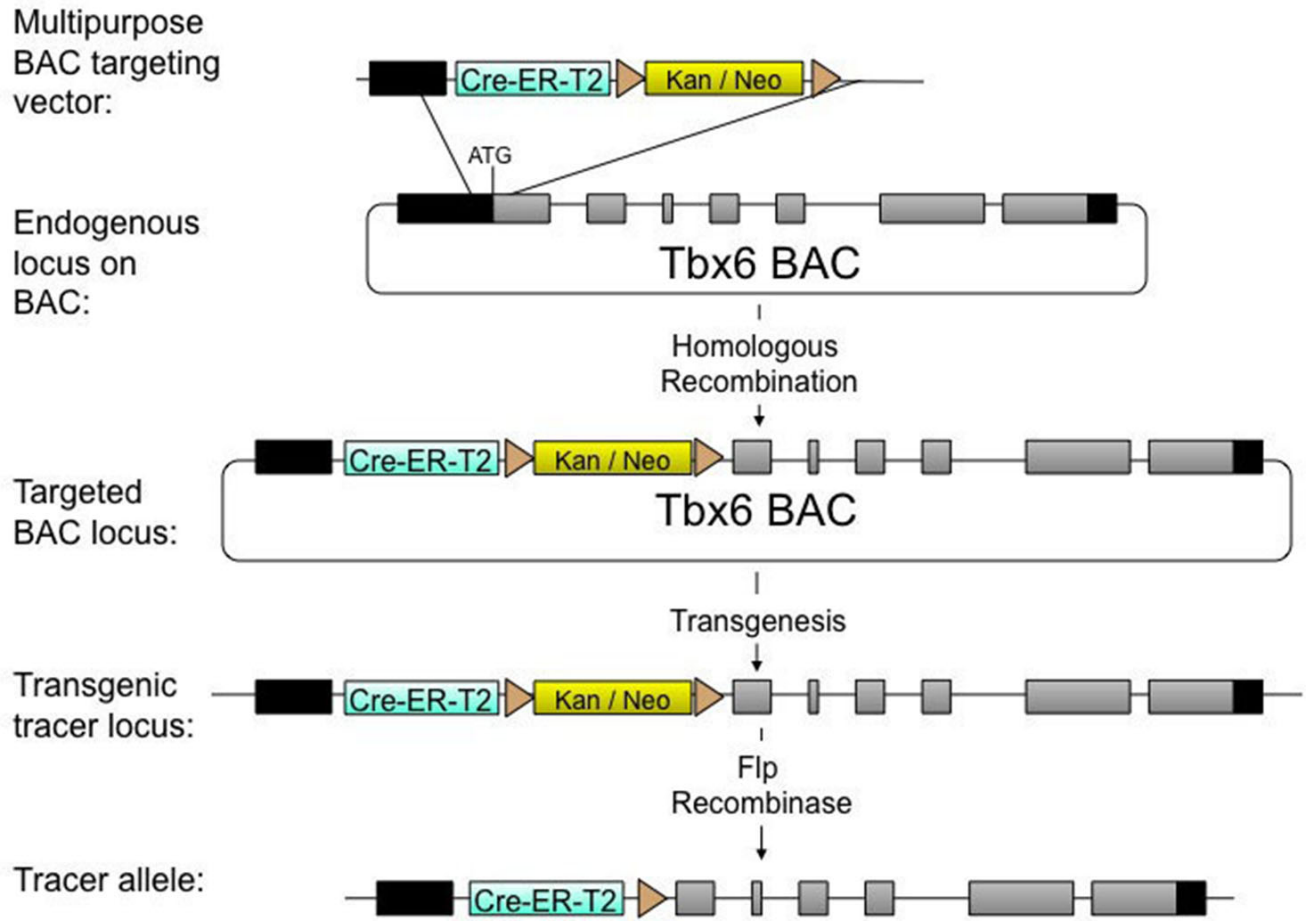
**Construction of an inducible, *cre* lineage-tracing allele under the control of *Tbx6* regulatory elements.** A multipurpose targeting vector containing homology to the endogenous *Tbx6* locus, an inducible *cre* gene, *cre-ERT2*, and a kanamycin/neomycin selection cassette surrounded by *frt* sites was targeted to a BAC containing the *Tbx6* locus. Following homologous recombination, the targeted BAC was injected into the pronuclei of mouse zygotes to produce random-insertion transgenic mice. These were bred with mice containing a Flpe transgene to eliminate the selection cassette, resulting in a *Tbx6* lineage-tracer allele inducible by tamoxifen.

A multipurpose bacterial artificial chromosome (BAC) targeting vector was used to create a lineage tracing allele of the *Tbx6* gene by targeting a *Tbx6-*containing BAC (Fig. 1). This allele contained a selection cassette flanked by FRT sites and a tamoxifen-inducible *creER^T2^* gene under the control of the endogenous *Tbx6* promoter. The modified BAC was then used to produce random insertion transgenic mice by pronuclear microinjection. Four *cre*-positive transgenic founders were bred to mice carrying a ubiquitous *flp* transgene to remove the selection cassette, producing mice carrying the lineage tracer allele (Fig. 1). Males were crossed with Cre-reporter mT/mG female mice and embryos were recovered at embryonic day (E)10.5 and examined under fluorescence microscopy to detect GFP fluorescence indicative of Cre activity. Descendants of two of the founders had low Cre activity and were not pursued; descendants of the other two had high levels of fluorescense in the *Tbx6* expression domain (not shown). The alleles in the latter two lines were named Tg(Tbx6-creERT2)^1Pa^ and Tg(Tbx6-creERT2)^2Pa^ and were tested further.

**Fig. 2.**
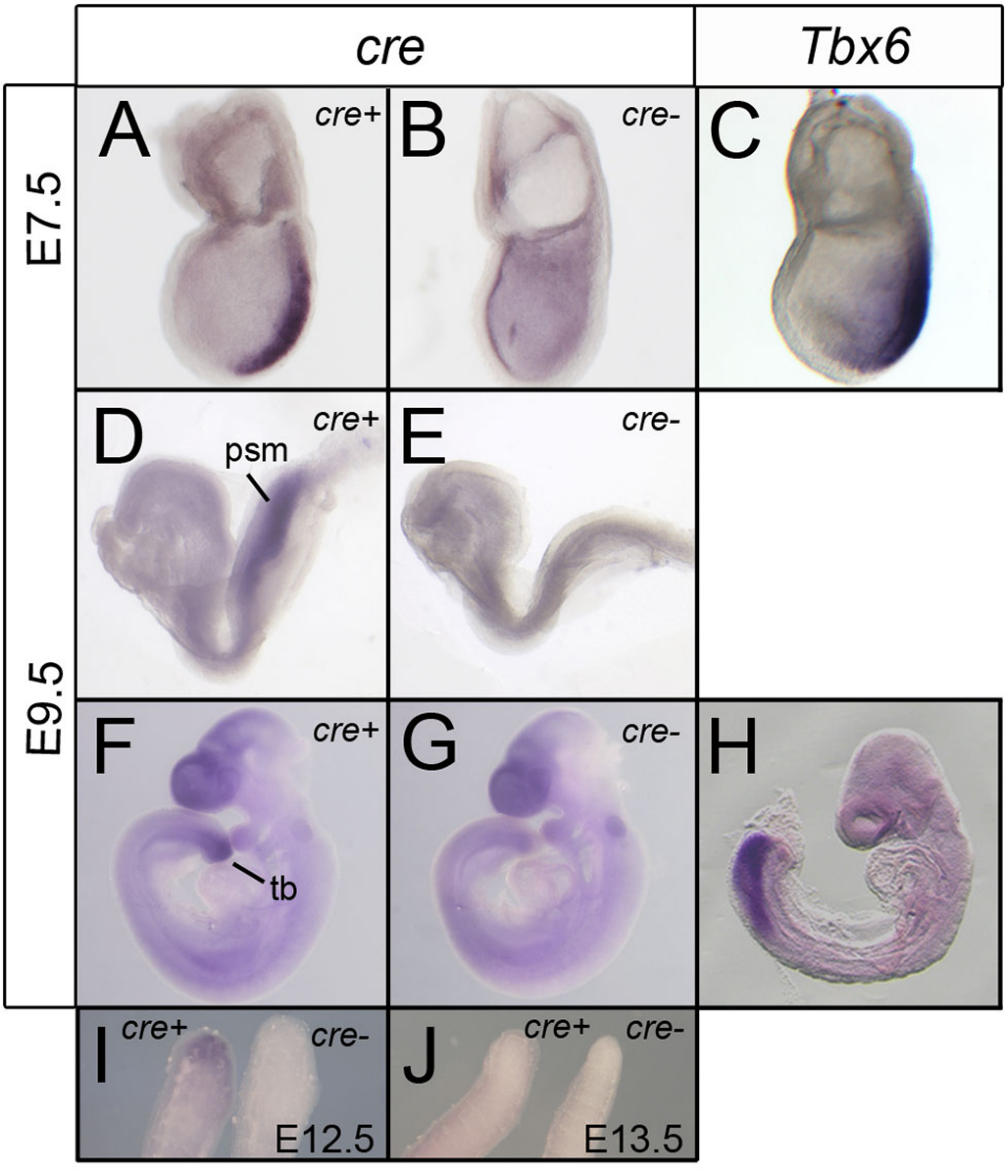
***Cre* expression in Tg(Tbx6-creERT2) transgenic embryos recapitulates endogenous *Tbx6* expression.** (A-H) Whole mount ISH for *Tbx6* (C,H) or *cre* (A,B,D-G,I,J) with transgene-negative (*cre*-) controls. At E7.5 *cre* expression is similar to *Tbx6* expression in the primitive streak, excluding the node, although *Tbx6* expression extends further into the lateral mesoderm. At E9.5, in embryos of different developmental stages, *cre* expression is limited to the presomitic mesoderm and tail bud although *Tbx6* expression extends somewhat further into the presomitic mesoderm than *cre* expression. There is high background in the forebrain and otic vesicle in both transgenic and non-transgenic embryos at the advanced E9.5 stage. (I,J) *cre* expression is present in the tip of the tail of an E12.5 transgenic embryo (left) compared to a nontransgenic control, but expression is lost by E13.5 (J) in most embryos. psm, presomitic mesoderm; tb, tail bud.

To determine whether the transgenes faithfully recapitulate *Tbx6* promoter activity both spatially and temporally, *cre* expression was documented by *in situ* hybridization (ISH) at several time points: E6.5, prior to the onset of *Tbx6* expression; E7.5, when *Tbx6* is expressed in the primitive streak; E9.5, when it is expressed in the presomitic mesoderm and tail bud; and E12.5 and E13.5, when *Tbx6* expression is extinguished (Chapman et al., 1996). For both transgenic lines, no expression was observed at E6.5 or in early stage E7.5 embryos, whereas some more advanced E7.5 embryos showed *cre* expression in the primitive streak and not in the node, as expected for endogenous *Tbx6* expression, but with less expansion into the lateral mesoderm (Fig. 2A-C, Table 1). At E9.5 all of the embryos genotyped by PCR as *cre-*positive showed *cre* expression by ISH in the tail bud and presomitic mesoderm comparable to but somewhat less extensive than the domain of *Tbx6* expression (Fig. 2D-H). For Tg(Tbx6-creERT2)^1Pa^, expression in the tail bud persisted at E12.5 and was gone in most embryos by E13.5 (Fig. 2I,J), whereas for Tg(Tbx6-creERT2)^2Pa^, *cre* was not expressed in the tail buds at E12.5. Thus, Tg(Tbx6-creERT2)^1Pa^ was chosen for further study as the timing of *cre* expression from the transgenic allele more closely recapitulates endogenous *Tbx6* expression, both temporally and spatially, with no evidence of ectopic expression (Fig. 2, Table 1).

**Table 1.**
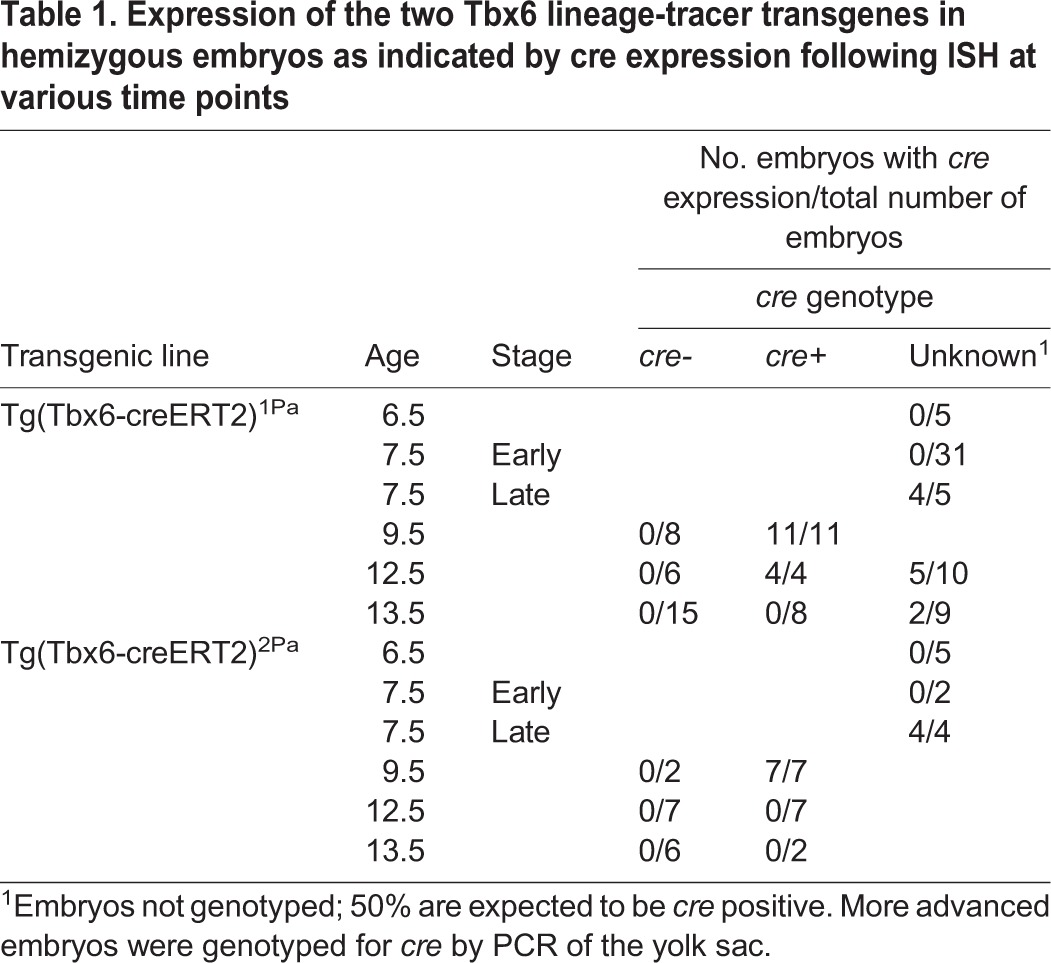
**Expression of the two Tbx6 lineage-tracer transgenes in hemizygous embryos as indicated by cre expression following ISH at various time points**

### Lineage tracing of cells that express *Tbx6* during normal development

To trace the lineage of cells and all their progeny that have expressed *Tbx6* at some point in development, hereafter referred to as Tbx6-creERT2-traced cells, males carrying the Tg(Tbx6-creERT2)^1Pa^ transgene and either wild type or heterozygous for *Tbx6^tm2Pa^* were crossed with R26R reporter females. Pregnant females were injected with tamoxifen between E5.5 and E9.5 to induce recombination in the reporter allele and embryos were recovered 2-5 days later to obtain a dynamic picture of the cohorts of Tbx6-creERT2*-*traced cells labeled at progressively later developmental stages (see Table 2 for the number of embryos evaluated at each time point by whole mount and/or sectioning). Recombination starts within 6-12 h of tamoxifen injection and continues for 36-48 h (Hayashi and McMahon, 2002; Joyner and Zervas, 2006), providing an effective labeling window from a single dose of tamoxifen of approximately 24-30 h. Thus, injection of pregnant females at E5.5, a day before *Tbx6* is expressed, is expected to capture a cohort of the earliest *Tbx6*-expressing cells with the labeling window extending to E7 (36 h post injection), and injection at E6.5 should label cells expressing *Tbx6* from approximately E6.75 to E8*.* Tamoxifen injections at 24 h intervals will therefore capture overlapping cohorts of Tbx6-creERT2-traced cells.

**Table 2.**
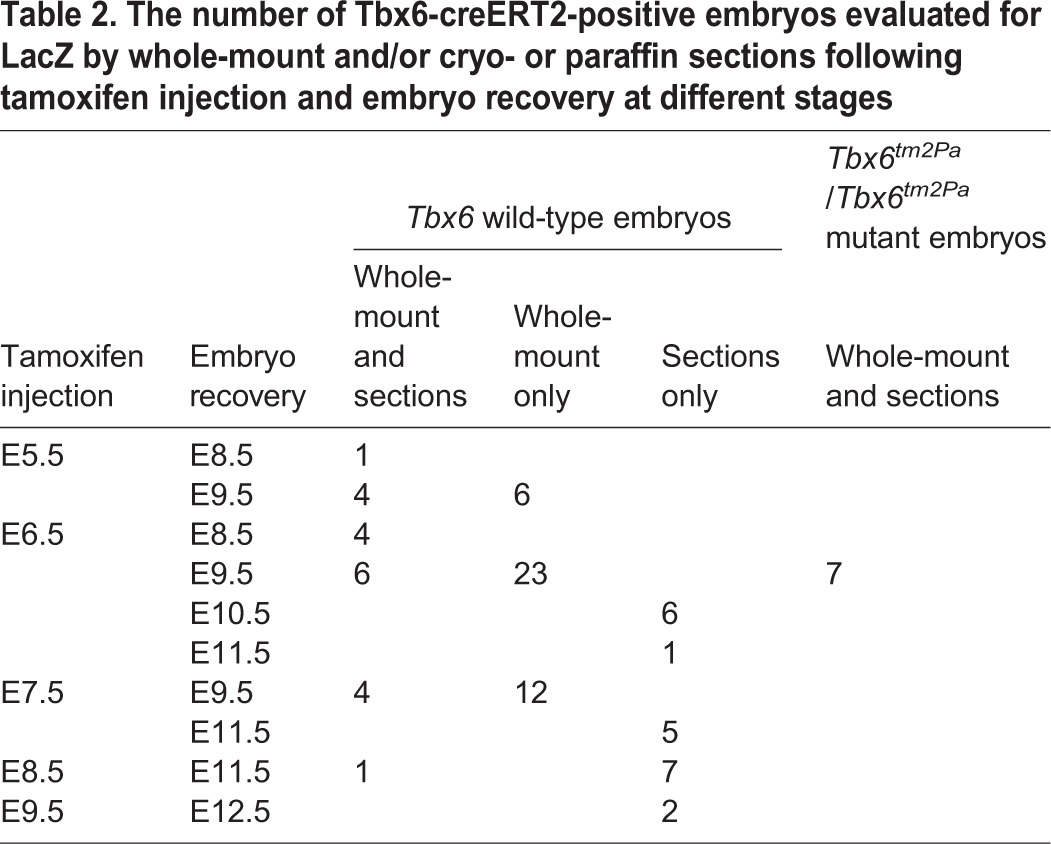
**The number of Tbx6-creERT2-positive embryos evaluated for LacZ by whole-mount and/or cryo- or paraffin sections following tamoxifen injection and embryo recovery at different stages**

**Fig. 3.**
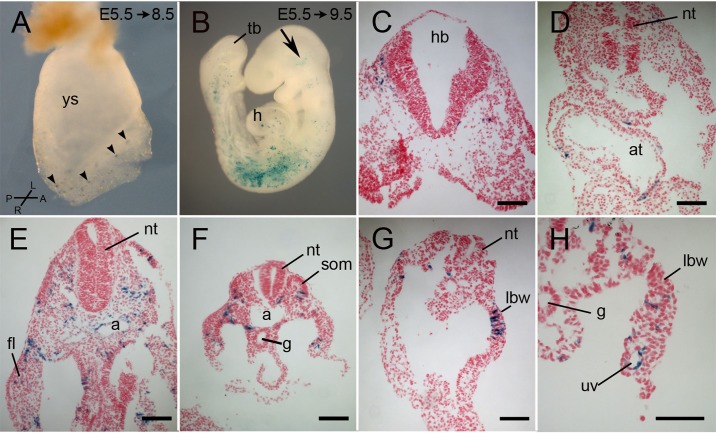
**Lineage tracing of a cohort of cells that expressed Tbx6-creERT2 between E6.5 and E7.0.** Pregnant Cre-reporter females were injected with tamoxifen at E5.5 and embryos were dissected at E8.5 (A) or E9.5 (B-H) and stained for lacZ. (A) An E8.5 unturned, whole mount embryos with scattered lacZ-positive cells (arrowheads) in the headfolds, lateral mesoderm, primitive streak and base of the allantois. (B) A whole mount E9.5 embryo of approximately 15-20 somites with lacZ-positive cells in the head (arrow), heart, and lateral mesoderm including somites, with lower concentrations in the rostral and caudal somites. (C-H) Representative transverse sections of E9.5 embryos showing lacZ-positive cells in the head mesenchyme at the level of the hindbrain (C), in the atrium of the heart (D), in the endothelium of the dorsal aorta and umbilical vein (E,H), somites, lateral body wall, forelimb bud, intermediate mesoderm and splanchnopleure mesoderm. a, aorta; at, atrium; fl, forelimb bud; g, gut; h, heart; hb, hindbrain; hf, headfold; lbw, lateral body wall; nt, neural tube; som, somite; tb, tail bud; uv, umbilical vein; ys, yolk sac. Compass in A refers only to panel A. Scale bars: 100 μm.

A few scattered blue cells representing the progeny of the earliest *Tbx6*-expressing cells were seen as far anteriorly as the headfolds in a headfold-stage embryo at E8.5, laterally along the axis of the embryo and posteriorly in the primitive streak and the base of the allantois (Fig. 3A, arrowheads). In embryos recovered a day later at 15-30 somites (Fig. 3B-H), labeled cells were concentrated in somites, with fewer labeled cells in the most rostral and most caudal somites. Labeled cells are located in the limb buds, when these are present, and in the lateral mesoderm extending posteriorly, but were not seen in the tail bud or presomitic mesoderm. Most embryos had some labeled cells in the head mesenchyme as far anteriorly as the level of the diencephalon, arranged in dorsal-ventral tracks (Fig. 3B, arrow), and about half of the embryos had a few labeled cells in the heart. Four of these embryos were sectioned and labeled cells were located in the head mesenchyme (Fig. 3C), heart (Fig. 3D), somites (Fig. 3E,F), intermediate mesoderm, somatopleure and splanchnopleure mesoderm derivatives of the lateral plate (Fig. 3E-H), and in the endothelium of blood vessels including the dorsal aorta and the umbilical vessels (Fig. 3E,H).

**Fig. 4.**
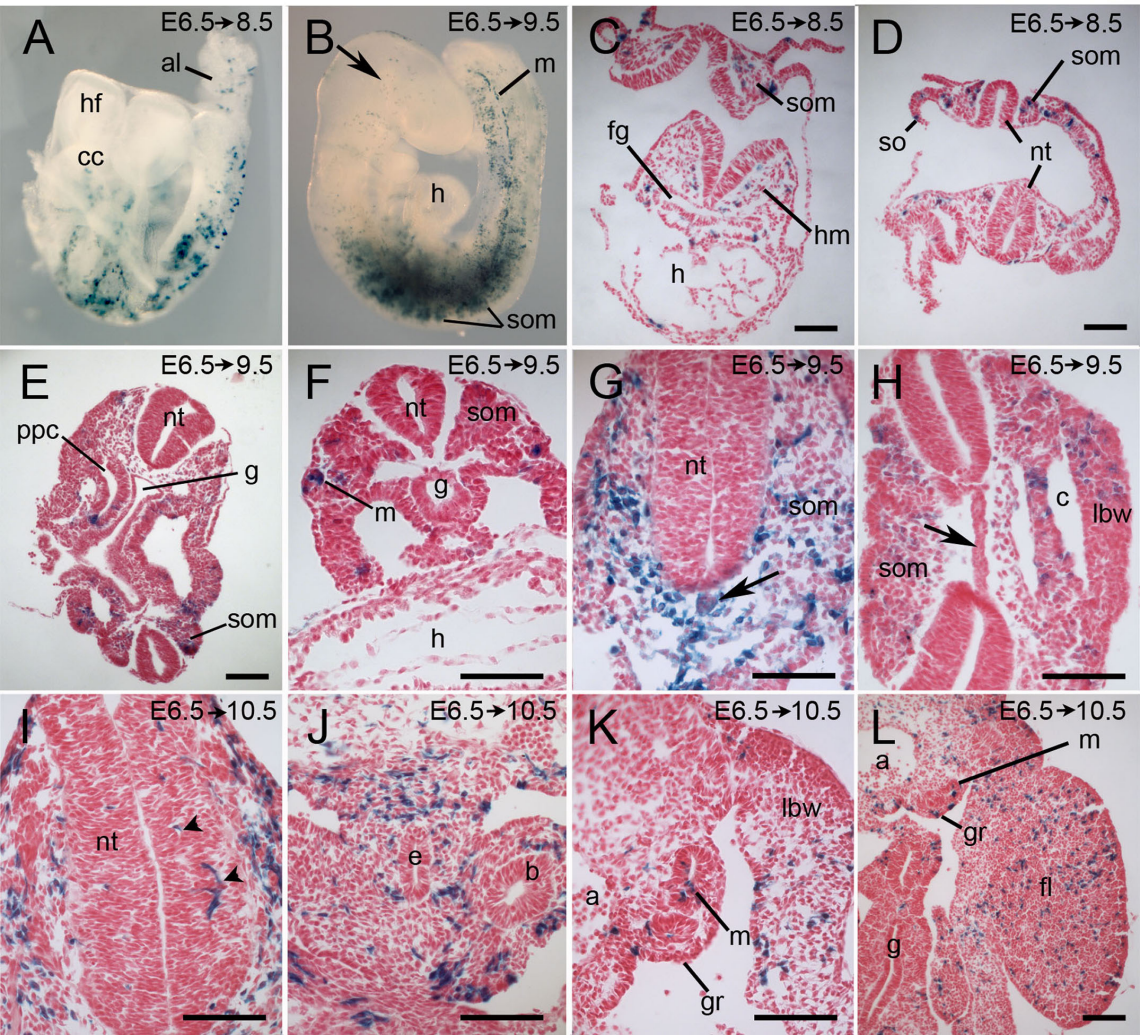
**Lineage tracing of a cohort of cells that expressed Tbx6-creERT2 between E6.75 and E8.** Pregnant Cre-reporter females were injected with tamoxifen at E6.5 and embryos were dissected 2-4 days later as indicated on each panel and stained for lacZ. (A) Unturned whole-mount E8.5 embryo with lacZ-positive cells in the allantois, cardiac crescent and laterally along the length of the embryo. (B) A whole-mount E9.5 embryo of approximately 20-25 somites with lacZ-positive cells in the head (arrow), somites, with higher concentration in somites 4-15, lateral body wall, and in a posterior lateral stripe marking the mesonephros. (C-L) Transverse sections of embryos showing representative tissues with labeled cells, notably head mesenchyme (C), heart (C), differentiating somites (C-I), mesonephric ducts (F,K), mesenchyme surrounding the esophagus and bronchi (J), in the lateral body wall and limb (H,K,L), lining of the coelom and pericardio-peritoneal canal (E,H), and genital ridge (K,L). A minority of embryos had labeled cells in the neural tube (arrowheads) (I) and notochord (black arrows in G and H show labeled and unlabeled notochord, respectively). Dorsal is to the top of each panel. a, dorsal aorta; al, allantois; b, bronchus; c, coelom; cc, cardiac crescent; e, esophagus; fg, foregut; fl, forelimb; g, gut; gr, genital ridge; h, heart; hf, head folds; lbw, lateral body wall; m, mesonephric duct; nt, neural tube; ppc, pericardio-peritoneal canal; so, somatopleure; som, somite. Scale bars: 100 μm.

The next cohort of Tbx6-creERT2-traced cells labeled by tamoxifen injection at E6.5 with a labeling window between E6.75 and E8 was sampled 2-5 days after injection. After 2 days, in unturned E8.5 embryos of 3-11 somites, labeled cells were seen in the head mesenchyme, cardiac crescent/heart, somites, along the lateral flanks of embryos extending posteriorly to the presomitic mesoderm and in the allantois (Fig. 4A,C,D). No label was seen in the node. Three days after injection, at E9.5 (12-30 somite stages), whole mounts revealed a scattering of labeled cells in the branchial arches and head (Fig. 4B, arrow), a few cells in the heart, and in the base of the allantois, while the main concentration of labeled cells was in the somites and lateral body wall, including the limb buds, and in the mesonephric ducts. Similar to the earlier labeled cell cohort, the most heavily labeled somites were in the caudal cervical and the thoracic region with fewer labeled cells in the rostral- and caudal-most somites and the presomitic mesoderm (Fig. 4B). Sections (Fig. 4E-H) revealed labeled cells in head mesenchyme and in all parts of the heart, a large contribution to somites, mesonephros, lateral body wall and coelomic epithelium (Fig. 4E-H), particularly around the pericardio-peritoneal canal. Notably, one of six embryos had extensive labeling of the notochord from the cervical region (Fig. 4G, arrow) and a second had several labeled cells in the posterior notochord, whereas the other four embryos showed no notochord labeling (Fig. 4H, arrow). 4-5 days after injection, labeled cells in the head were concentrated around the otic vesicle; they were also located in the branchial arches, heart, differentiating somites (Fig. 4I), limb buds and lateral body wall (Fig. 4K,L), endothelium of the aorta, coelomic epithelium, mesenchyme surrounding the gut and bronchi (Fig. 4J,L) and in the genital ridge and mesonephric tubules (Fig. 4K,L). In five of the seven embryos sectioned, a few labeled cells were seen within the hindbrains or cervical neural tube (Fig. 4I, arrowheads), but none were seen in the notochords.

**Fig. 5.**
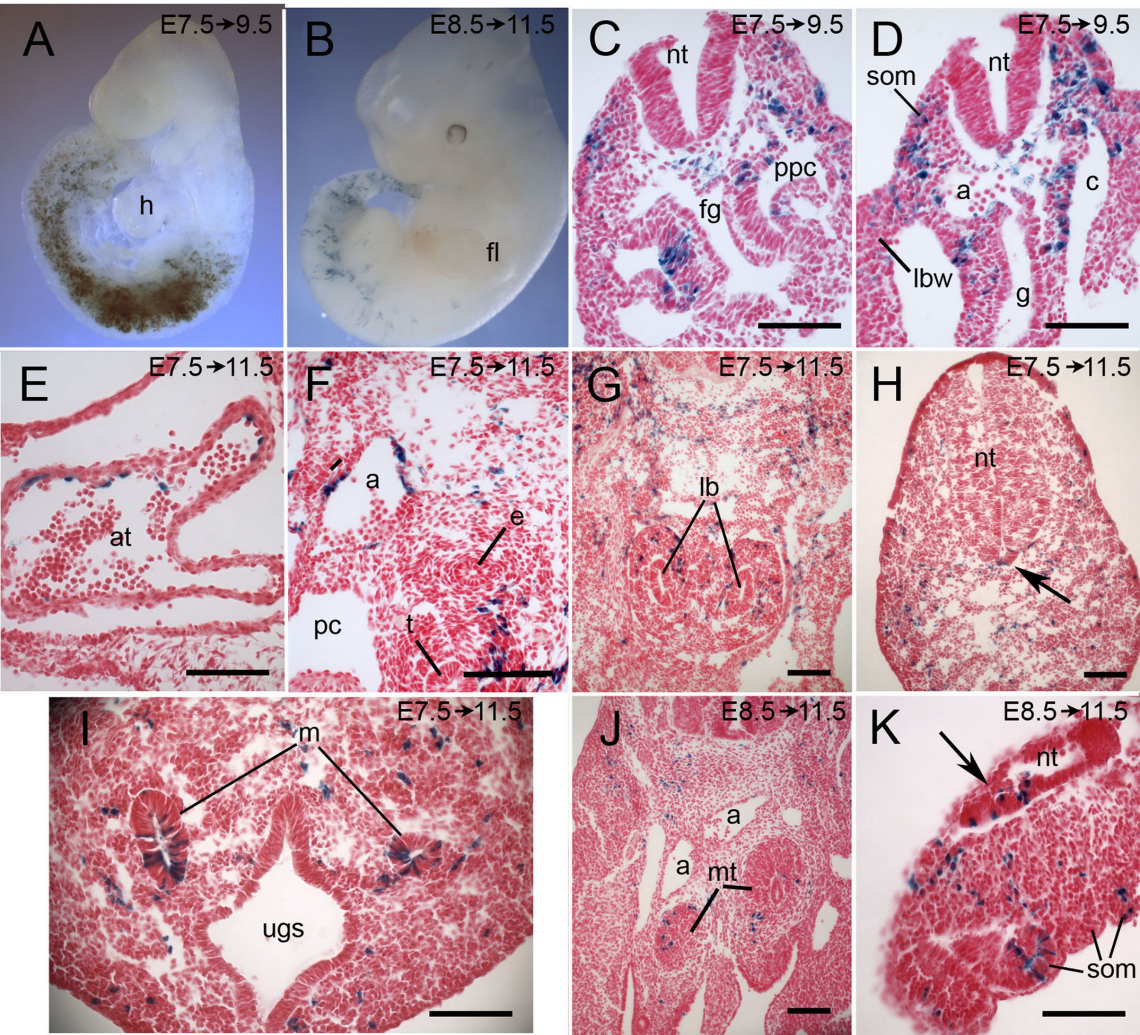
**Lineage tracing of cohorts of cells that expressed Tbx6-creERT2 between E7.75 and E9 or between E8.75 and E10.** Pregnant Cre-reporter females were injected with tamoxifen at E7.5 (A,C-I) or E8.5 (J,K) and embryos were dissected 2-4 days later as indicated on each panel and stained for lacZ. (A) A whole-mount embryo labeled at E7.5 with the bulk of the lacZ-positive cells in the somites and lateral mesoderm. (B) A whole-mount embryo labeled at E8.5 with a pronounced posterior shift in the distribution of labeled cells. (C-K) Transverse sections of embryos showing representative tissues with labeled cells, notably somites (C,D,K), lining of the coelom and pericardio-peritoneal canal (C,D), endothelium of the heart and aorta (D-F), mesenchyme surrounding the lung buds and trachea (F,G), mesonephric tubules and metanephric blastema (I,J), and tail mesenchyme (H,K). In a small number of embryos, labeled cells were seen in the posterior neural tube (arrow in H, in the floorplate of the tail neural tube; arrow in K, in tangential section of tail neural tube). a, dorsal aorta; at, atrium; c, coelom; e, esophagus; fg, foregut; fl, forelimb; g, gut; h, heart; lb, lung buds; lbw, lateral body wall; m, mesonephric duct; mt, metanephric blastemal; nt, neural tube; pc, pericardial cavity; ppc, pericardio-peritoneal canal; som, somite; t, trachea; ugc, urogenital sinus. Scale bars: 100 μm.

The cohort of cells labeled at E7.5 with a labeling window between E7.75 and E9 was sampled 2 or 4 days later at 20-35 somite stages and showed a similar distribution to the previous cohort but with a posterior shift in the concentration of labeled cells such that there were fewer in the head, heart and rostral somites (compare Figs 4B and 5A). Labeled cells were seen in head mesenchyme, differentiating somites and their derivatives, mesenchyme of the body wall, mesenchyme surrounding the viscera (Fig. 5F,G), limb buds, heart (Fig. 5E), aorta (Fig. 5F), mesonephric ducts (Fig. 5I), tail bud mesenchyme (Fig. 5H) and in a few cells of the posterior notochord in one of the nine embryos sectioned. As observed for the earlier cohort of labeled cells, there were a few scattered labeled cells in the hindbrain or cervical neural tube in three embryos and, in addition, one embryo had a few labeled cells in the floorplate of the neural tube in the tail region just caudal to the hind limb (Fig. 5H, arrow).

*Tbx6-*expressing cells labeled at E8.5 (labeling window between E8.75 and E10) and recovered 3 days later at 30-40 somite stages showed a pronounced posterior shift compared with earlier cohorts (Fig. 5B) with labeled cells only as far rostral as the mid-thoracic region. Labeled cells were seen in the tail mesenchyme and somites (Fig. 5K), hind limbs, mesonephric tubules and metanephric primordia (Fig. 5J), the genital papilla, and in the neural tube in the tail of two of the eight embryos analyzed (Fig. 5K, arrow). Progeny of *Tbx6-*expressing cells labeled at E9.5 and recovered three days later were further restricted to mesenchyme of the tail only (not shown).

### Lineage tracing in Tbx6 homozygous mutant embryos

**Fig. 6.**
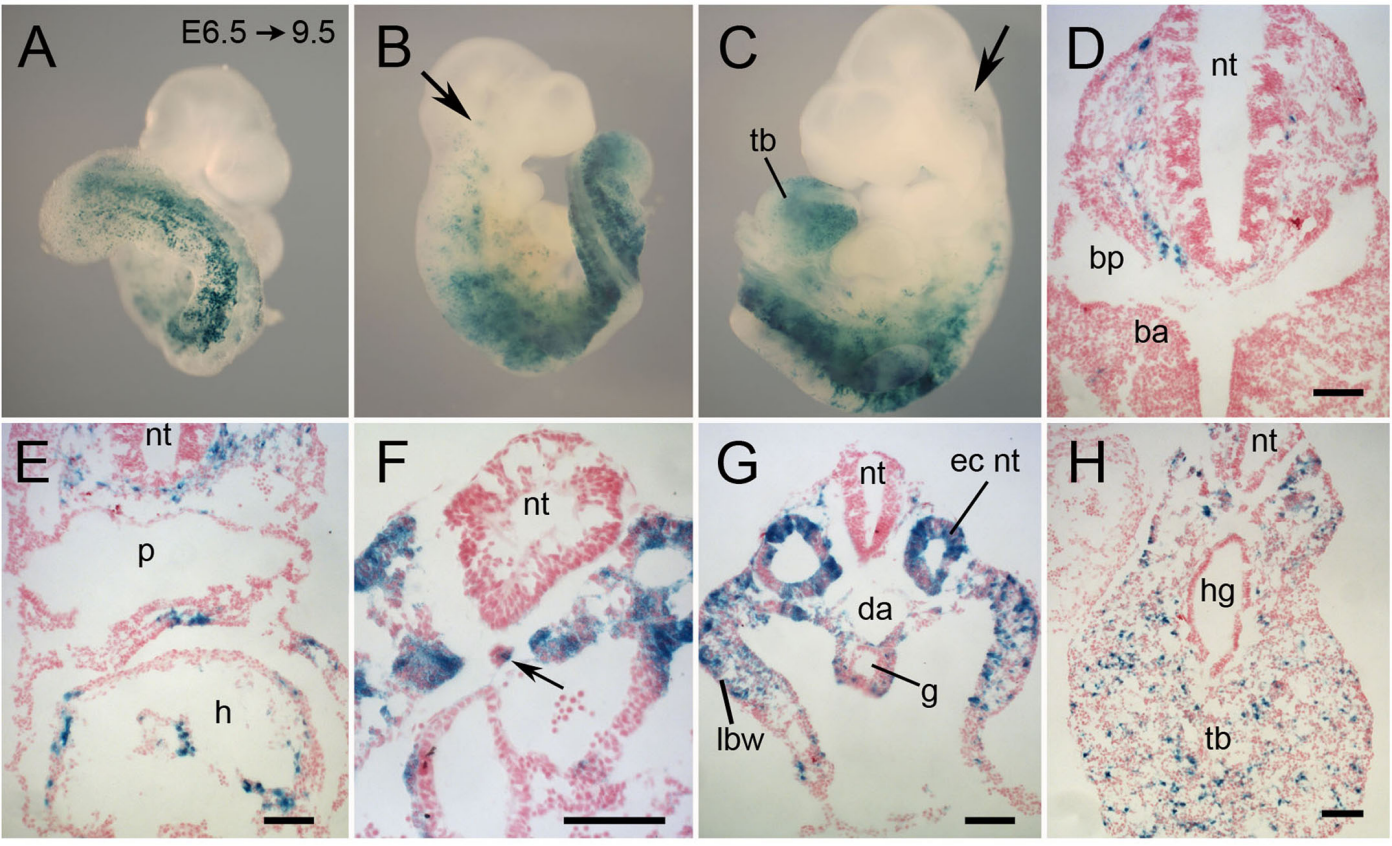
**Lineage tracing in *Tbx6* homozygous mutant embryos of a cohort of cells that expressed Tbx6-creERT2 between E6.5 and E7.** Cre-reporter females heterozygous for a *Tbx6* null allele were crossed with *Tbx6* heterozygous males and were injected with tamoxifen at E6.5; embryos were recovered at E9.5 and stained for lacZ. (A-C) Whole-mount embryos of different developmental stages with lacZ-positive cells in the head region (arrows in B,C), in the irregular anterior somites, in the trunk corresponding to the ectopic neural tubes characteristic of the *Tbx6* homozygous phenotype and in the expanded tail bud. The exaggerated twisting of the torso is a feature of the mutant phenotype. (D-H) Representative transverse sections showing lacZ-positive cells in the head mesenchyme (D,E), heart (E), ectopic neural tubes (F-H), notochord (1/7 embryos; arrow in F), lateral body wall (G), mesenchyme surrounding the gut (G) and throughout the mesenchyme of the expanded tail bud (H). ba, branchial arch; bp, branchial pouch; da, dorsal aorta; ec nt, ectopic neural tube; g, gut; h, heart; hg, hindgut; lbw, lateral body wall; nt, neural tube; p, pharynx; tb, tailbud. Scale bars: 100 μm.

It was of interest to determine whether the lack of Tbx6 alters the distribution of presomitic mesoderm cells and also to determine the origin of the tissue that makes up the ectopic neural tubes in homozygous mutant embryos. Because mutant embryos die shortly after E9.5, lineage tracing was limited to a single cohort of cells labeled by tamoxifen injections at E6.5 with recovery at E9.5. Homozygous mutants, recognized by their expanded tail bud and the lack of posterior somites (Chapman and Papaioannou, 1998), showed patterns of LacZ expression in whole mounts that resembled wild-type embryos (compare Fig. 6A-C with 4B) with the exception that there was a greater concentration of labeled cells in the expanded mutant tail buds (Fig. 6A-C,H). Sections of seven mutant embryos revealed labeled cells in the head mesenchyme (Fig. 6D,E), the heart (Fig. 6E) and endothelial lining of blood vessels, in the limb buds, lateral body wall, intermediate mesoderm, and splanchnopleure surrounding the gut (Fig. 6F,G), the coelomic epithelium, mesonephros and notably, the ectopic neural tubes (Fig. 6F,G). One embryo also had a few labeled cells in the notochord (Fig. 6F). Gut endoderm and axial neural tube had no labeled cells.

## DISCUSSION

### Normal lineage of cells that have expressed *Tbx6*

Temporal and spatial regulation of *Tbx6* expression is complex and is controlled by multiple enhancers and repressors (White et al., 2005, 2003). Lopez and Fan (2012) made use of a genomic region containing an enhancer to produce several random-insertion, inducible *Tbx6-cre* transgenes, one of which was expressed robustly in the presomitic mesoderm and in newly formed somites, in addition to several areas of ectopic expression. The regulatory region used in this transgene is missing a somite silencer element required to restrict expression to the presomitic mesoderm, resulting in somite expression, and also drives expression with a lag of one day compared to endogenous *Tbx6* expression (White et al., 2005). Thus, while useful for conditional manipulation of gene function in the PSM and somites, it is not suitable for expression-lineage tracing.

Based on our experience with a ‘knock-in’ reporter that similarly did not fully recapitulate normal *Tbx6* expression (Hadjantonakis et al., 2008 and personal observations), we chose to make a *Tbx6-*cre reporter using BAC transgenesis in order to preserve all regulatory elements controlling *Tbx6* expression. By documenting *cre* expression and also using reporter mice to assay Cre activity in the progeny of two transgenic founders with the BAC transgene, we showed that Tg(Tbx6-creERT2)^1Pa^ provides an accurate reporter of *Tbx6* promoter activity as judged by the close temporal and spatial correlation between *cre* and endogenous *Tbx6* expression with several caveats that may limit expression of *cre* to a subset of *Tbx6* expresssing cells: first, the timing of Tg(Tbx6-creERT2)^1Pa^ expression may be slightly delayed compared with *Tbx6*, as expression was not seen until the late bud stage at E7.5, whereas *Tbx6* can be detected at the early bud stage (Nowotschin et al., 2012); secondly, as seen from ISH, *cre* expression appears less extensive than *Tbx6* expression in the presomitic and lateral mesoderm. A further limitation of this reporter is that following administration of the maximum dose of tamoxifen compatible with embryo survival, it appears that only a subset of the *Tbx6-*expressing cells undergoes Cre-dependent excision as all areas of lacZ expression subsequently appear mosaic. Thus, we can use the lineage tracer to determine what tissues Tbx6-creERT2-traced cells contribute to, but cannot say whether a tissue is composed entirely of cells that have expressed *Tbx6.*

Endogenous *Tbx6* expression is first detected by ISH in the early primitive streak and in the paraxial mesoderm surrounding the streak between E7 and E7.5, extending from just caudal to the node to the base of the allantois. Between E8.5 and E9.5, *Tbx6* is expressed in the unsegmented, presomitic mesoderm of the posterior region surrounding the caudal end of the neural plate and in mesoderm surrounding the posterior end of the hindgut. Expression is extinguished as somites form but persists in the unsegmented presomitic mesoderm and tail bud for as long as new somites are being formed (Chapman et al., 1996; Nowotschin et al., 2012). As expected from this expression pattern, the lineage of the earliest Tbx6-creERT2-traced cells closely matches the cell fate of the primitive streak and paraxial mesoderm as determined by classical fate mapping studies (Smith et al., 1994; Wilson and Beddington, 1996). Following tamoxifen injection at E5.5 and E6.5, which should capture the earliest *Tbx6*-expressing cells, labeled cells are detected rostrally in the head mesenchyme and caudally in the extraembryonic allantoic mesoderm, presumably deriving from the rostral and caudal regions of the primitive streak, respectively. Cells were also located throughout the paraxial mesoderm, in cardiac mesoderm, somites, intermediate mesoderm and in the mesoderm layers of the lateral plate somatopleure and splanchnopleure, all areas known to be derived from different regions of the early primitive streak (Smith et al., 1994; Wilson and Beddington, 1996). Relatively few labeled cells were present in the presomitic mesoderm and tail bud, in line with the expectation that following a limited labeling period, most labeled cells move out of the presomitic mesoderm as they differentiate. When chased to later time points, these early cohorts of cells made contributions to derivatives of virtually all mesoderm tissues, contributing to the limb buds, somites, including the most rostral somites, and mesonephric ducts, metanephros, blood vessels, and, notably, the notochord. Descendants of cohorts of cells labeled at later developmental stages showed very similar patterns of deployment with the exception that the bulk of labeled cells was located progressively more caudally.

In addition to the mesoderm derivatives, Tbx6-creERT2-traced cells were detected in sections of the hindbrain or rostral neural tube of 8/38 embryos injected with tamoxifen at E6.5 or later and recovered at E10.5 or later. Several observations point to these cells being invading vascular cells. First, no Tbx6-creERT2-traced cells are present in the neural tube of any embryos recovered prior to E10.5, indicating that these cells have migrated into the neural tube, and secondly, their morphology is generally not typical of neural cells but closely resembles that of invading vascular cells, as described by Nakao et al. (1988) a process that begins at E9.5. Three additional embryos injected with tamoxifen at E7.5 or E8.5 and recovered at E11.5 had labeled cells in the neural tube or floorplate of the neural tube in the tail region, but these cells were less typical of vascular cells and may represent a late contribution of Tbx6-creERT2-traced cells to the posterior neural tube.

### Tbx6-creERT2-traced cells in *Tbx6* homozygous mutants

Our results help resolve several outstanding questions regarding the phenotype of homozygous *Tbx6* mutants. Vascular abnormalities were assumed to be the cause of death of *Tbx6* mutants due to the presence of multiple hematomas (Chapman and Papaioannou, 1998). Our results showing contributions of Tbx6-creERT2-traced cells to the endothelium of major vessels and to the heart support this hypothesis. Similarly, the expansion of the tail bud in mutants and in chimeras (Chapman et al., 2003) has been attributed to a failure of ingressing cells to migrate away from the primitive streak. Comparison between the cohorts of cells labeled at E6.5 and followed 3 days later in wild-type and mutant embryos supports the notion that the wild-type cohort has moved out of the tail bud, but that a large proportion of the mutant cohort has remained in the tail buds of the mutants. Finally, our results show that the ectopic neural tubes of *Tbx6* mutant embryos are derived from the Tbx6-creERT2-traced cells that have ingressed through the streak, and not from ectoderm or the axial neural tube.

Another feature of the *Tbx6* mutant phenotype is the formation of 8-12 abnormally segmented rostral somites that are progressively more abnormal caudally to the level of the forelimb bud, where the ectopic neural tubes begin (Chapman et al., 2003; Chapman and Papaioannou, 1998). The lineage results indicate that Tbx6-creERT2-traced cells do contribute to all of the rostral somites, however, even in the cohort of cells labeled at the earliest stages of *Tbx6* expression, there is a rostral-to-caudal gradient of low-to-high contribution of cells to these somites. This supports the idea that only some of the cells of the most rostral somites have expressed *Tbx6*, perhaps protecting them from the mesoderm to neural transdifferentiation that occurs more caudally (Takemoto et al., 2011). This interpretation is in accordance with the results of chimera experiments in which *Tbx6* mutant embryonic stem (ES) cells can contribute to rostral but not caudal somites in mutant/wild-type chimeras (Chapman et al., 2003). In these same chimera experiments, mutant ES cells were largely excluded from the gut, leading to the hypothesis that *Tbx6* may be involved in gut formation. However, there is no evidence from this lineage study that Tbx6-creERT2-traced cells contribute to the gut.

The presence of labeled cells in the notochord of three wild-type embryos and one mutant embryo was unexpected on the basis of fate maps that indicate that the notochord derives from the node (Beddington, 1994; Sulik et al., 1994; Wilson and Beddington, 1996), and the fact that *Tbx6* expression has never been documented in the node (Chapman et al., 1996; Hadjantonakis et al., 2008). However, *Tbx6* mutant embryos have abnormalities in the node, particularly in the nodal cilia, resulting in randomized left/right axis determination (Hadjantonakis et al., 2008). The lineage analysis indicated here would imply that at least some cells of the node expressed *Tbx6* at some point in their development. Cell lineage studies of early stage embryos, prior to node formation, indicate that some precursors of the node/notochord reside in the rostral portion of the E6.7 prestreak region or E7.5 rostral early streak (Lawson et al., 1991; Tam and Beddington, 1987). As cilia are assembled on node precursor cells prior to their emergence on the embryo's surface and the formation of the node (Lee and Anderson, 2008), this could explain the abnormalities in *Tbx6* mutants. It should be noted however, that the notochord was labeled in only 4/48 embryos in the entire study. Thus, if this explanation is correct, the Tbx6-creERT2-traced cells may contribute only a small proportion of cells to the node even though cilia of all the node cells are affected in mutants.

These results provide novel information through GIFM on the fate of cells that express the T-box transcription factor, Tbx6, and on how the multiple abnormalities caused by mutations in *Tbx6* are mediated. They also point to many areas colonized by Tbx6-creERT2-traced cells where no mutant phenotypes have yet been reported, but that may provide areas for future study of *Tbx6* function.

## MATERIALS AND METHODS

### Generation of Tbx6-creERT2 transgenic mice

Using the Sv129 BAC bmq-279H8 (The Wellcome Trust Sanger Institute, Hinxton, UK) as a template, two regions of homology, upstream and downstream of the *Tbx6* translational start site were cloned into a multipurpose BAC targeting vector along with an optimized, inducible *cre-ER^T2^* with a poly-adenylation site (gift from T. Ludwig, Columbia University Medical Center, New York, USA) and a kanamycin/neomycin selection cassette cloned using pL451 as a template. The final construct contained the homologous arms flanking the translational start site of *Tbx6*, *cre-ER^T2^* with its own translational start site and poly-adenylation signal, and a kanamycin/neomycin selection cassette driven by a dual bacterial/mammalian promoter flanked by FRT sites in between these two arms (Fig. 1; see Table S1 for primers used).

Established protocols (Washkowitz and Shaywitz, 2007) were used to target this construct to the *Tbx6*-containing BAC, bmq-279H8. Briefly, we transformed an erythromycin-resistant plasmid containing an arabinose-inducible version of the recombinase system from bacteriophage lambda into the BAC-containing bacteria. This bacterial strain was grown overnight at 37°C, subcultured into 50 ml of LB medium at a concentration of 1:100 and grown to an OD600=0.2. At this point, 20% L(+) Arabinose (Sigma #10839) 1:200 was added to the culture and it was incubated at 37°C to a final OD600 of 0.6-0.8. The bacteria were then collected in sterile water and electroporated at 2.3 kV, 200Ω, 25 μF with the linear fragment of the targeting construct generated by digestion with Sac2. The bacteria were allowed to recover for 75 min at 37°C and then plated on LB+Cm (20 μg/ml)+Kan (12.5 μg/ml). Colonies were picked and tested for the BAC insertion using PCR with primers that encompassed a region outside of the homolgous arms of the targeting vector as well as a region inside. Positive colonies were phenol-chloroform extracted, transformed into fresh bacteria and tested for kanamycin and chloramphenicol resistance and erythromycin sensitivity. These bacteria were grown and the BAC was isolated and injected into the pronucleus of C57BL/6 embryos by the Columbia University Medical Center Transgenic Facility.

### Animals and genotyping

All animal experiments were carried out according to protocols approved by the Columbia University Medical Center Institutional Animal Care and Use Committee. C57BL/6 potential transgenic founder animals were genotyped by PCR for the presence of *cre*. For removal of the kanamycin/neomycin cassette, founders were mated with ROSA26::FLPe transgenic mice (Farley et al., 2000) to obtain *cre-*positive, *neo-*negative, *flpE*-positive animals. These were mated with ICR random bred mice (Taconic, Germantown, NY, USA) for maintenance of the transgenes and in subsequent generations, mice without the *flpE* transgene were selected for breeding. PCR was done according to standard conditions using the primers indicated in Table S1.

Cre expression in the founder transgenic lines was tested by mating *cre*-positive animals with random bred ICR females and recovering embryos for *in situ* hybridization with a 1000 bp *cre* probe. More advanced embryos were genotyped for *cre* by PCR of the yolk sac. Cre enzymatic activity was also tested by mating *cre*-positive animals with the Cre reporter strains Gt(ROSA)^26Sortm4(ACTB-tdTomato,-EGFP)Luo^/J (The Jackson Laboratory, Bar Harbor, ME, USA; called mT/mG) or B6.129S4-Gt(ROSA)26Sor^tm1Sor^/J (The Jackson Laboratory; called R26R). Pregnant females were injected intraperitoneally with tamoxifen (20 mg/ml; 5-7 mg/mouse) in sunflower seed oil (Sigma, 8001-21-6), which has the effect of translocating Cre-ERT2 to the nucleus where it excises the reporter gene, and embryos were examined under fluorescence microscopy or were subjected to X-Gal staining for LacZ.

### *In situ* hybridization (ISH)

Embryos were fixed overnight at 4°C in fresh 4% paraformaldehyde (PFA) (Sigma, 30525-89-4), then dehydrated and stored at −20°C in 100% methanol. Embryos were rehydrated for whole-mount ISH performed as described by Wilkinson (1992). ISH was performed for *cre-ER^T2^* mRNA using a DIG-labeled single stranded RNA probe, developed using a standard labeling assay kit (Roche Applied Science, Indianapolis, IN, USA; Cat. No. 11-277-073-910).

### Lineage tracing using lacZ

For lineage tracing, mice carrying a *cre* transgene and wild type or heterozygous for a null allele *Tbx6^tm2Pa^* (Hadjantonakis et al., 2008) were mated with R26R reporter females, some of which were also heterozygous for *Tbx6^tm2Pa^* for the collection of homozygous mutants that were recognized morphologically. The day of the copulation plug was considered E0.5. Pregnant females were injected at different stages of gestation with tamoxifen (most received 5 mg/mouse with a few in early experiments receiving 6 or 7 mg/mouse), and in later experiments in combination with progesterone (2 mg/mouse), which greatly reduced the rate of embryo resorption caused by tamoxifen alone (Joyner and Zervas, 2006). Embryos were collected 2-5 days later, genotyped for *cre* by PCR, fixed in 4% PFA at 4°C for 1 h, and *cre*-positive embryos were either stained as whole mounts with X-Gal prior to cryosectioning or paraffin embedding and sectioning, or were cryosectioned and then stained for X-Gal (Lobe et al., 1999). Nuclear fast red counterstain was used for all sections. For cryosectioning, embryos were placed in 30% sucrose (Sigma, 57-50-1) at 4°C, overnight or longer and then washed in optimum cutting temperature medium (OCT) for 1 h at 4°C followed by rapid freezing in fresh OCT. Blocks were stored in 2-methylbutane (Sigma, 78-78-4) at −80°C until sectioning at 4-10 µM. Embryos for paraffin embedding were dehydrated, embedded and sectioned at 8-10 µM. The numbers of *Tbx6* wild-type and mutant embryos examined at each time point by whole mount and/or sections is shown in Table 2.
